# A next generation vaccine against human rabies based on a single dose of a chimpanzee adenovirus vector serotype C

**DOI:** 10.1371/journal.pntd.0008459

**Published:** 2020-07-15

**Authors:** Federico Napolitano, Rossella Merone, Adele Abbate, Virginia Ammendola, Emma Horncastle, Francesca Lanzaro, Marialuisa Esposito, Alessandra Maria Contino, Roberta Sbrocchi, Andrea Sommella, Joshua D. Duncan, Jospeh Hinds, Richard A. Urbanowicz, Armin Lahm, Stefano Colloca, Antonella Folgori, Jonathan K. Ball, Alfredo Nicosia, Benjamin Wizel, Stefania Capone, Alessandra Vitelli

**Affiliations:** 1 ReiThera Srl, Rome, Italy; 2 Wolfson Centre for Global Virus Infections, University of Nottingham, Nottingham, United Kingdom; 3 School of Life Sciences, University of Nottingham, Nottingham, United Kingdom; 4 Department of Molecular Medicine and Medical Biotechnology, University Federico II, Naples, Italy; 5 GSK Vaccines, Rockville, Maryland, United States of America; US Department of Agriculture, UNITED STATES

## Abstract

Rabies, caused by RNA viruses in the Genus Lyssavirus, is the most fatal of all infectious diseases. This neglected zoonosis remains a major public health problem in developing countries, causing the death of an estimated 25,000–159,000 people each year, with more than half of them in children. The high incidence of human rabies in spite of effective vaccines is mainly linked to the lack of compliance with the complicated administration schedule, inadequacies of the community public health system for local administration by the parenteral route and the overall costs of the vaccine. The goal of our work was the development of a simple, affordable and effective vaccine strategy to prevent human rabies virus infection. This next generation vaccine is based on a replication-defective chimpanzee adenovirus vector belonging to group C, ChAd155-RG, which encodes the rabies glycoprotein (G). We demonstrate here that a single dose of this vaccine induces protective efficacy in a murine model of rabies challenge and elicits strong and durable neutralizing antibody responses in vaccinated non-human primates. Importantly, we demonstrate that one dose of a commercial rabies vaccine effectively boosts the neutralizing antibody responses induced by ChAd155-RG in vaccinated monkeys, showing the compatibility of the novel vectored vaccine with the current post-exposure prophylaxis in the event of rabies virus exposure. Finally, we demonstrate that antibodies induced by ChAd155-RG can also neutralize European bat lyssaviruses 1 and 2 (EBLV-1 and EBLV-2) found in bat reservoirs.

## Author summary

Rabies still remains a neglected zoonosis after a long history of vaccination. Considering the severity of the disease and its continued high incidence in low-income countries, the development of a next generation vaccine is warranted. We utilized a group C, replication-defective chimpanzee adenovirus vector to develop a novel vaccine against rabies. Mice vaccinated with ChAd155-RG survived after rabies infection and non-human primates injected with a single dose of this vaccine developed strong and durable neutralizing antibody responses which could be effectively boosted with a licensed vaccine, demonstrating the compatibility of the novel vectored vaccine with the current post-exposure prophylaxis in the event of rabies virus exposure. Importantly, we show that ChAd155-RG induced neutralizing antibodies can neutralize also lyssavirus species (EBLV-1 and EBLV-2) found in bat reservoirs. These studies paved the way to the clinical testing of the ChAd155-RG based rabies vaccine as a single-dose, low cost, preventative rabies vaccine candidate.

## Introduction

Rabies is an acute, progressive encephalitis caused by a *Lyssavirus*, usually after the bite of an infected mammal. This zoonosis is the most fatal of all infectious diseases and still remains a major public health problem in developing countries, causing the death of tens of thousands of people each year. In the second WHO report on neglected diseases [1], methodical analysis of the country-specific official published figures of rabies occurrence indicated that the reported incidences may actually be an underestimation of the total disease burden by as much as >100-fold or more in many countries, as most of rabies-related deaths occur at home in communities away from hospitals. Often rabies cases are misdiagnosed as other neurological diseases, even in developed countries. Most rabies deaths, with more than half of them in children, result from dog bites and occur among low-income families in Asia and Africa. Multiple mammalian species serve as reservoirs of *Lyssaviruses*, particularly among the Carnivora and Chiroptera.

The most widely circulating and relevant for human health of the *Lyssavirus* genus is the *Rabies virus* (RABV), but all *lyssaviruses* cause the same disease: rabies [2]. Apart from Antarctica, all other continents are enzootic for rabies, but without necessarily the presence of RABV, per se. For example, *Australian Bat Lyssavirus* (ABLV), with reservoirs among fruit bats of the Genus *Pteropus*, is the only documented lyssavirus causing fatal rabies cases among humans, domestic animals and wildlife in Australia. Regardless of viral species, all lyssaviruses share a common morphology, phylogeny and a relatively simple RNA genome that encodes five structural proteins. Of these, the virus glycoprotein is the only target for virus neutralizing antibodies (VNA), which provide full protection against RABV challenge [3].

Safe and efficacious human and veterinary rabies vaccines are available. The development of the first rabies vaccine, long before recognition of the nature of viruses, was developed in 1885 by Louis Pasteur and represented a milestone in public health history. For more than 70 years, only vaccines containing nerve tissues were available. A major step forward was the development, in the 1960s, of ‘modern’ rabies vaccines, prepared from RABV grown in cell culture and inactivated. These were safe and highly effective in preventing rabies [4]. Uniquely among vaccines, human rabies biologics are usually administered after viral exposure. Post-exposure prophylaxis (PEP) is possible because the exposure event, usually a dog bite, is easily identifiable, and the incubation period is long enough for vaccination to induce a protective immune response before RABV reaches the central nervous system. After exposure to RABV, vaccines have to be given 2 to 5 times by intradermal (ID) or intramuscular (IM) injection over a course of 7 days or 3 weeks, depending on the recommended protocols. In situations of severe bite exposure, vaccines have to be combined with RABV-specific immune globulins (RIG), infiltrated into the bite site (WHO position paper 2018). In part, the high incidence of human rabies, in spite of effective vaccines, is linked to the lack of compliance with the administration schedule, due to ignorance of appropriate PEP and the cost and limitation in supply of affordable biologics.

Pre-exposure prophylaxis (PrEP) consists of 2 to 3 ID or IM injections at days 0, 7, or 0, 7 and 21 or 28. Modern PrEP can be a viable strategy in diminishing the number of rabies deaths, especially those resulting from unapparent or unreported exposures, and delayed or incomplete PEP. However, PrEp is largely underused as a strategy to protect children in high-risk areas [5]. For these reasons, rabies still remains a neglected disease after a history of 125 years of vaccination. Nevertheless, WHO recognizes that children living in rabies-affected areas are at particular risk and encourages the implementation of carefully designed studies on the feasibility and cost-effectiveness and long-term impact of incorporating rabies vaccines into the immunization programs of infants [6] [7]. Considering the severity of rabies and its continued high incidence in developing countries, development of novel vaccines is warranted. Such a vaccine would have to provide sustained protection, preferably after a single dose application, be easy to administer and economically affordable.

Chimpanzee-derived adenovirus-vectored (ChAd) vaccines are an attractive platform technology for induction of immune responses, circumventing the potential problem of pre-existing anti-vector antibody to human adenovirus serotypes [8]. There is ample clinical experience with chimpanzee derived, replication-defective adenoviruses, which demonstrates that they are safe and highly immunogenic [9]. An especially attractive feature of this vaccine platform is that immune responses are sustained due to very low-level persistence of the vectors in a transcriptionally active form [10]. In addition, methods to stabilize such vectors to allow for their use in countries where cold chains are not available have been developed [11].

We describe here the construction and preclinical evaluation of two novel chimpanzee-derived replication-defective adenoviral vectors, ChAd155 and ChAd83 belonging to group C and group E, respectively, encoding the rabies G protein. Since the vector belonging to group C showed superior immunogenicity in mice, it was selected as the vaccine candidate for further *in vivo* studies.

The immune correlate of protection from rabies is well characterized and consists of serum virus neutralizing antibody (VNA) titer exceeding 0.5 international units per milliliter (IU/ml). This threshold is also considered as the endpoint for clinical efficacy [12]. We demonstrate that a single-dose of the vectored vaccine ChAd155-RG can induce serum VNA titers well above the seroconversion threshold in vaccinated non-human primates, at comparable levels to a full three-dose schedule of a licensed inactivated rabies vaccine. Moreover, ChAd155-RG-induced VNA can be boosted six months after vaccination with one dose of a licensed vaccine, demonstrating the compatibility of the novel vectored vaccine with the current post-exposure prophylaxis in the event of rabies virus exposure. Finally, by pseudotyping of a murine leukemia virus retroviral vector with various lyssavirus glycoproteins we show that ChAd155-RG induced neutralizing antibodies can cross-neutralize also *Lyssavirus* species found in bat reservoirs. This next-generation rabies vaccine candidate is currently being tested in a Phase 1 clinical trial to establish its suitability as a low cost preventative rabies vaccine.

## Methods

### Chimpanzee adenovirus vector construction and production

ChAd155 and ChAd83 vectors were derived respectively from wild-type chimpanzee Ad type 155 genome and wild chimpanzee Ad type 83 genomes, isolated from healthy captive chimpanzees using standard procedures [8]. The viral genomes were then cloned into plasmid vectors and fully sequenced. The E1 and E4 regions were deleted from the viral genomes and the replication-defective viral vectors were propagated in PROCELL92 cells [13]. Deletion of the E1 region was from bp 449 to 3529 for ChAd155, and from bp 451 to 3413 for ChAd83. The E4 region was deleted from bp 34731 to 37449 for ChAd155 and from bp 33747 bp 3613 for ChAd83. and replaced with the Ad5E4orf6Based on hexon protein sequence comparisons, ChAd155 and ChAd83 represent subgroup C and E human adenoviruses, respectively. Indeed, ChAd155 and ChA83 genome sequence analysis shows very high similarity with other subgroup C and E adenoviruses, suggesting that they can use the same receptor (Coxsackievirus and Adenovirus Receptor, CAR) to enter the host cell. In both vectors, the transgene expression cassette has been inserted by homologous recombination in the E1 region. The human CMV promoter drives the transcription of the transgene and the bovine growth hormone (BGH) poly(A) sequence is downstream to the transgene stop codon. In some constructs, the woodchuck hepatitis virus posttranscriptional regulatory element (WPRE) was inserted upstream of the polyadenylation sequence. ChAd155-RG and ChA83-RG vectors were grown in PROCELL92 cells [13], purified by cesium chloride gradients and stored in buffer A195 [14]. Viral particle (vp) measurements of adenovirus stocks were made by real time PCR on the viral genome.

### Rabies vaccine antigen design

In order to increase the cross-protective breadth of the vaccine, the sequence of the G glycoprotein cloned into the ChAd vector represents the ‘medoid’ sequence selected amongst 2060 aligned G protein sequences from phylogroup I and II lyssaviruses taken from the NCBI nr database, i.e. the G sequence with the highest average percent of amino acid identity to all other G protein sequences. The selected G sequence (NCBI accession number AGN94271, isolate A11_4583) shares ≈ 94% average sequence identity with the other G proteins. Another approach consisted of including the nucleoprotein N, in addition to G, in the vaccine antigen. For the N protein the same methodology was applied selecting AGN72885 (isolate 9319IRA), which had an average sequence identity of 98.3% to 2541 N proteins. Determination of the medoid sequences was performed in R (http://www.R-project.org) using R libraries seqinR [15] and peplib (https://github.com/whitead/peplib)

### Assessment of antigen expression in infected cells

#### Western blot analysis

A549 or HeLa cells were infected with ChAd155-RG, ChAd155-RGN, ChAd155-RNG and ChAd83-RG vectors at multiplicity of infections (MOI) of 50, 250, 1250 vp/cell. Extracts were prepared 48 hours after infection using TEN buffer (20 mM Tris pH 7.5, 150 mM NaCl, 1 mM EDTA pH 8, 1% Triton X100 and protease inhibitors). Nuclei and cell debris were spun out by centrifugation at 7,500×*g*, 60 minutes at 4°C. Glycerol (10% final concentration) was added to the supernatants before storage at −20°C. Expression of the antigen in the cell extracts was assessed by running the samples in reducing SDS–PAGE and probing with a rabbit polyclonal serum against rabies G protein (Alpha Diagnostic).

#### FACS analysis

Human MRC5 cells were infected with ChAd155-RG vector. 48 hours post-infection the cells were detached and washed with phosphate-buffered saline (PBS). Cells were then stained with anti-rabies glycoprotein antibody mAb 8727 (Millipore) and FITC-conjugated anti-mouse secondary antibody (eBioscience). Cells were first gated on their forward- and side-scatter profiles. The gated cells were subsequently differentiated by their forward-scatter profile and fluorescent signal.

### Animals and immunogenicity studies

Animal protocols describing in details the experimental procedures were reviewed and approved by the institutional ethics committee to ensure that the experiments were in compliance with their respective authorized projects as defined by the Directive 2010/63/EU and national regulations (Italian Legislative Decree 26/2014, French decree No. 2013–118).

#### Mice

six-week-old female CD1 or BALB/c mice purchased from Envigo, were acclimatized and housed in individually vented cages at the Plaisant animal facility (Castel Romano, Rome, Italy). The work was performed under Italian ministry of health authorization number 1065/2015-PR. Animal handling procedures (immunization and bleed) were performed under isofluorane anesthesia. Animals were divided into experimental groups of 6, 8 or 10 mice each depending on study, and immunization was performed via intramuscular injection (quadriceps), 50μl volume in each leg (100μl total volume). Mice were bled via retro-orbital sinus and serum was obtained by coagulation and centrifugation for rabies virus neutralizing antibody measurements; mice were euthanized by cervical dislocation and splenocytes were isolated for T cell response measurements.

#### Rabbits

To compare immunogenicity of a single dose of ChAd155-RG or RABIPUR, a study in rabbits was conducted at Citoxlab France (project #4048-as per Directive 2010/63/EU and ethical committee approval filed n.03721). Ten male KBL New Zealand White rabbits of 3–4 months of age and with body weight in a range between 2.5 and 3.5 kg were acclimatized for 7 days while ascertaining their good health. The animals were individually housed in noryl cages containing at least one object for environmental enrichment, within a dedicated rabbit unit. The animals were then allocated to two study groups according to a computerized stratification procedure based on body weight. Each group received either one IM administration of 5 x 10^10^ vp of ChAd155-RG (half of the intended human dose) or half human dose of RABIPUR (500 μL), respectively. The injection site was the anterior right thigh, clipped free of hair. The animals were monitored for local reactions, body weight and food consumption, and were bled (approximately 4ml) from appropriate arteries to isolate serum at baseline, week 1, 3, 8 and 12. At the end of study the animals were euthanized by exsanguination following deep anesthesia by IV injection of pentobarbital.

#### Macaques

The study was conducted at Aptuit srl (Verona, Italy) under approval of the internal Aptuit Committee on Animal Research and Ethics and under authorization issued by the Italian Ministry of Health (Italian Ministry of Health Authorization nr. 984/2015-PR). Fifteen male purpose-bred Cynomolgus monkeys (*Macaca fascicularis*) originating from Mauritius were housed in groups of 7 or 8 in six communicating stainless steel cages, with constant access to environmental enrichment devices within a dedicated non-human primate unit. The animals, approximately 3 years of age and 3 to 4 Kg of body weight at study start were assigned to three equivalent study groups of five animals each according to their body weight. The vaccination schedule is reported in Table 1.

**Table 1:** NHP vaccination schedule.

group (n = 5)	vaccines	dose^a^	schedule (weeks/days)
1	ChAd155-RG ChAd155-RG	5 x 10^10^ vp 5 x 10^10^ vp	w0 w48
2	ChAd155-RG RABIPUR ChAd155-RG	5 x 10^10^ vp ½ human dose 5 x 10^10^ vp	w0 w24 w48
3	RABIPUR 3X	½ human dose	days 1-7-21

^**a**^ vp: viral particles

The animals were vaccinated intramuscularly (left deltoid or alternating deltoids in the case of repeated administrations close in time) with: a single dose of 5x10^10^vp ChAd155-RG (group 1 and 2) or three doses (d0, 7, 21) of RABIPUR (group 3). For both vaccines, half of an intended human dose was used, and an injection volume of 0.3 (ChAd155-RG) or 0.5ml (RABIPUR) was given. Animals in group 2 received a booster injection of RABIPUR at week 24, and a final immunization with ChAd155-RG was given to groups 1 and 2 at week 48. Animals vaccinated with RABIPUR were followed up to six months, while animals in group 1 and 2 that received the vectored vaccine were followed up to one year. At the end of follow up (week 24 for group 3 and week 52 for groups 1 and 2) the animals were euthanized by intravenous injection of barbiturate sodium thiopental 200 mg/ml (0.75ml/Kg) and sodium heparin 5000 Iu/ml (0.2 ml/Kg) followed by exsanguination. Vaccinations and bleeding by femoural vein or artery occurred in conscious animals, unless sedation by IM injection of 0.1 ml medetomidine and 0.2 ml ketamine was deemed necessary. Blood volumes (3.5 ml no anticoagulant for serum, 20 ml in Litium heparin for PBMC isolation) and frequency were adequate to animals of this age and body weight.

### Immunological analyses

#### Fluorescent Antibody Virus Neutralization (FAVN)

Rabies Virus Neutralizing Antibodies (VNA) in serum of immunized mice, rabbits and macaques was measured by means of a validated, WHO approved test well established at IDEXX BioResearch—Vet Med Labor GmbH. Briefly, sera were diluted in D-MEM-10% FCS medium according to the following dilution scheme: log dilution 0,48–0,95–1,43–1,91–2,39–2,86–3,34–3,82–4,29–4,77–5,25–5,73 for an endpoint titration, in 96 well plates. A fixed amount of 100 TCID50 rabies virus (‘challenge virus standard’ [CVS-11] strain adapted to cell culture) was added to each well and plates were incubated at 35–37°C with 5% CO_2_ for 1 hour. BHK-21 cells were added to each well and microplates are incubated for 48 hours at 35–37°C. The controls plate contains also replica wells of cells only-no virus, and of virus only-no serum. Cell layers were probed with FITC anti-rabies, and wells were qualitatively scored as “positive” or “negative” depending on the presence or absence of one or more fluorescent cell. The serum titer is the dilution at which 100% of the virus is neutralized in 50% of the wells (log D50). This titer is then expressed in IU/ml by comparing it with the neutralizing dilution of the OIE reference serum of dog origin under the same experimental conditions.

**Rapid Fluorescent Focus Inhibition Test (RFFIT)** was performed by mixing different dilutions of mouse sera with a constant amount of rabies virus, [CVS-11] strain adapted to cell culture, for a short time before adding the mixture to cells, in which the virus can replicate. After incubation for ~20 hours, cells were fixed and stained to detect rabies virus production, by reading the slide using a fluorescent microscope. A total of 20 microscopic fields were read for each serum dilution, and compared against a control slide containing reference serum and virus dilutions. The number of infected fields for each serum dilution was used to determine the rabies virus neutralizing titer. The serum neutralization end-point titer is the highest serum dilution in which there is a 50% reduction in the number of fluorescing foci. The Reed-Meunch formula is applied to calculate the difference between the logarithm of the starting dilution and the logarithm of the 50% end-point dilution. The 50% end-point titers of the reference serum and the mouse serum are then compared to calculate the IU/ml.

#### ELISA with mouse sera

Rabies antibodies in immunized mice sera was tested by means of two different ELISA. A commercially available ELISA kit (PLATELIA RABIES II kit from Bio-Rad) which utilizes a rabies PV strain glycoprotein antigen extracted from virus membrane, and was used according to manufacturer’s instructions. Titers are expressed as equivalent units (EU)/ml, which correlates with International units (IU)/ml.

#### IFN-γ *ex vivo* ELISpot

rabies-specific T cell responses in mice splenocytes and in macaque PBMCs were determined by a standard IFNγ ELISpot assay. Briefly, 96-well plates (MSIP S4510 Millipore) were coated with 10μg/ml of anti-mouse or anti-monkey IFNγ antibody (U-CyTech Utrecht, the Netherlands) and incubated overnight at 4°C. Macaque PBMCs were thawed and incubated overnight at 37° C to allow apoptotic cells to die. After blocking the plates, cells were plated in duplicate wells at 200,000 PBMCs per well. Mouse splenocytes were plated in duplicate at 200,000 and 400,000 cells per well. Cells were stimulated overnight with RG peptide pools (1μg/ml for splenocytes and 3μg/ml for PBMCs as final concentration) consisting of 15-mer sequences with 11–amino acid overlaps to cover the sequence of rabies glycoprotein. DMSO (Sigma) and concanavalin A (Sigma) were used as negative and positive controls, respectively. Plates were developed with biotinylated anti-mouse or anti-monkey IFN-γ antibody (U-CyTech Utrecht, The Netherlands), conjugated streptavidin–alkaline phosphatase (BD Biosciences, San Jose, CA) and with 1-Step NBT/BCIP solution (Thermo Fisher Scientific, Rockford, IL). Plates were analyzed by Immunospot S6 Ultimate UV image analyzer (CTL Europe GmBh). A positive ELISpot response was considered to be at least 50 specific spots/million splenocytes or 40 specific spots/million PBMCs on at least one peptide pool and three times the number detected in the negative control wells.

#### Intracellular cytokine staining on frozen PBMC

1 × 10^6^ cells/well were seeded in a 96-well tissue culture plate with rabies peptide pools in the presence of Brefeldin A (Sigma, 10μg/ml final concentration) and costimulatory antibody (anti-CD28 and CD49d) according to the manufacturer’s instructions. DMSO and phorbol 12-myristate 13-acetate (Sigma)/ionomycin (Sigma) were used as negative and positive controls, respectively. The plate was incubated 5 hours (5% CO2, 37°C). After stimulation the cells were stained with L/D-Violet dye (Life Technologies Ltd.) in PBS solution. After washing, the cells were stained with the following antibodies: CD3e APC (clone SP34-2), CD4 PerCpCy5.5 (clone L200) and CD8a PerCPCy5.5 (clone RPA-T8). All antibodies were purchased from BD Biosciences. Cells were then permeabilized with Cytofix/Cytoperm (BD) and stained in Perm/Wash (BD) with anti-IFNγ FITC (clone MD-1, UCyTech). Acquisition was performed on the day of staining on a CytoFlex flow cytometer (Beckman Coulter); at least 30,000 CD8 events were collected per sample. Data analysis was performed using CytExpert software.

### Rabies challenge study

The rabies challenge study was performed at Lyssa LLC, Cumming, GA, USA. Groups of 10 females, 4–6 weeks old CD1 mice each received a single IM (gastrocnemius, 50μl volume) injection of escalating doses (10^5^ to 10^8^ vp) of ChAd155-RG, or 10^8^ vp of a ChAd155 encoding a rabies-unrelated antigen (ChAd155-mock, negative control). A control group received the standard 3 injection schedule of the commercially available cell culture-derived vaccine (CCV) RABIPUR (positive control) given at 1/10_th_ of the human dose. Mice were bled via retro-orbital sinus at various time points throughout the study (day 0, 14, 28, 56, 67). The serum VNA titers were measured by a standard Rapid Fluorescent Focus Inhibition Test (RFFIT). At 60 days after vaccination, the animals were challenged IM with 1x10^4^ TCID/mouse of RABV, with an anticipated ~ 100% mortality based upon previous in vivo titration. The challenge virus was a Street RABV (variant Ps P4, isolated from a fatal human case associated with exposure to a rabid bat) isolated at Lyssa LLC. Mice were observed daily for further 30 days, and brains were collected from mice showing signs of illness and euthanized by carbon dioxide inhalation as well as from all survivors at the study conclusion (study day 90) for detection of rabies virus antigens by the direct fluorescent antibody (DFA) test.

### Biodistribution study in rats

A biodistribution study upon single intramuscular administration of ChAd155-RG was conducted at Citoxlab France (project #2958-as per Directive 2010/63/EU and ethical committee approval filed n.03797) under OECD GLP conditions. Sprague-Dawley rats (24 males and 24 females) obtained from Janvier (France) were acclimatized for 8 days and were of approximately 7 weeks of age at study start (mean weight 312g for male and 213g for females). The animals were group-housed (maximum three and from the same experimental group) in polycarbonate cages with environmental enrichment in a barriered rodent unit. Four groups of 5 male and 5 female Sprague-Dawley rats each received a 100μl 2.3x10^10^ vp dose of ChAd155-RG by intramuscular injection (right quadriceps). Immunizations occurred under isofluorane anesthesia. In addition, 1 group of 4 males and 4 females received NaCl 0.9% as control. The animals were checked daily for mortality and clinical signs, and weekly for body weight and food consumption. At 24 hours, 7, 28 or 48 days after treatment (respectively at Day 2, Day 8, Day 29 and Day 49 of the study), groups of male and female animals were deeply anesthetized by an intraperitoneal injection of pentobarbital sodium, bled (at least 0.6 ml in K_2_ EDTA from the abdominal aorta) and euthanized by exsanguination. One animal/sex from saline control group was bled and euthanized at the same time-points as the ChAd155-RG treated animals. Designated organs (injection site-right quadriceps muscle, inguinal, right iliac and popliteal lymph nodes, brain, hearth, kidneys, liver, spleen, lungs, ovaries or testis) were collected and tissue specimens were snap frozen. DNA was extracted from all tissue samples collected 24 hours and 7 days after the injection with NucleoSpin Tissue or blood Quick Pure kits (Macherey-Nagel) and analyzed for ChAd155-RG genome by a validated qPCR method using primers and probes detecting the hCMV promoter region: forward primer: 5’- CATCTACGTATTAGTCATCGCTATTACCA -3’; reverse primer: 5’- GACTTGGAAATCCCCGTGAGT -3’; internal Taqman probe: 6-FAM- 5’- ACATCAATGGGCGTGGATAGCGGTT -3’. When samples were negative for the test item DNA at 2 consecutive time points, samples from the corresponding tissues at the following time points were neither extracted nor analyzed.

### Production and titration of pseudoviruses

Pseudovirus production and pseudovirus-based neutralisation assays (PBNA) were performed at the University of Nottingham, UK. Lyssavirus glycoprotein sequences (rabies virus strain CVS-11 (EU352767), European bat lyssavirus 1 (EU352768), European bat lyssavirus 2 (EU352769), Mokola virus (HM623780), West Caucasian bat virus (WCBV) (EF614258) and Ikoma virus (IKOV) (JX193798) cloned into the pI.18 plasmid vector were kindly provided by Dr Edward Wright, University of Sussex [16]. The VSV glycoprotein was cloned into the pI.18 plasmid to serve as a non-specific virus control. HEK-293T cells were seeded at a density of 1.2 x 10^6^ into 100mm dishes 24 hours prior to transfection. 2 μg each of the MLV gag/pol construct phCMV5349 and the firefly luciferase reporter construct pTG126 were transfected along with the lyssavirus glycoprotein constructs in Optimem reduced serum media using 24 μl polyethylenimine [17]. Media was replaced with DMEM 6 hours after transfection. Pseudovirus-containing supernatant was harvested and filtered through a 0.45 μm filter 72 hours post-transfection and stored in aliquots at -20°C. Pseudovirus infectivity was determined prior to freezing. 100 μl of pseudovirus preparations were added to 2 x 10^4^ BHK-21 cells on a white 96 well plate. After 4 hours, 150 μl fresh DMEM was added to the cells and they were incubated for a further 72 hours. The media was then removed and the cells lysed using 50 μl cell lysis solution (Promega). 50 μl of luciferase substrate (Promega) was added to cells immediately prior to measuring luminescence with a Fluostar Omega luminometer at an optical gain of 2000 for undiluted pseudoviruses and 3600 for diluted pseudoviruses. Pseudovirus preparations used in PBNA were diluted to produce a luminescence output >30,000 RLU when measured at a gain of 3600.

### In vitro PBNA

Macaque serum samples were heat inactivated then diluted in a 5-fold serial dilution in PBS. 25 μl of each serum dilution was added to 75 μl of pseudovirus and incubated for 1 hour at room temperature prior to addition to BHK-21 cells. After 4 hours, 150 μl of DMEM containing 1% penicillin/streptomycin was added to the cells. The cells were incubated for 72 hours before being lysed and luminescence measured. The infectivity of the pseudoviruses was calculated as a percentage relative to uninhibited pseudovirus and ΔEnvelope controls. Best-fits curves and IC50 values were calculated using GraphPad Prism using a non-linear regression model (inhibitor versus response with a four parameter variable slope). The mean IC50 values for each immunogen serum against each virus were compared using 2-way ANOVA and Sidak’s multiple comparison test.

### Statistical analysis

GraphPad Prism version 6 for Windows (GraphPad Software, San Diego, California, USA) was used for graphs and statistical analysis. Since immunogenicity data mostly showed non-Gaussian distribution, as per the D’Agostino-Pearson omnibus K2 normality test, non-parametric tests were used throughout unpaired two tailed Mann-Whitney test to compare two groups, or Kruskal-Wallis with Dunn’s Multiple Comparison post hoc test to analyze multiple groups. Percent survival (Kaplan-Mayer) curves data were analyzed by Log-rank (Mantle-Cox) test. Two-way ANOVA was used to analyze effect of treatment, time and their interaction for rabbit single dose study. For comparison of pseudovirus neutralization assays, data were first analyzed for Gaussian distribution using the D’Agostino-Pearson test. Single point neutralization data were normally distributed and therefore plotted as means with standard deviation, with differences between treatments compared using one-way ANOVA followed by multiple comparison tests. Consolidated and normalized neutralization curves were fitted using 4 parameters, variable slope non-linear regression analysis. A p value < 0.05 was considered significant. Only statistically significant results were reported in the figures, *p ≤ 0,05; **p ≤ 0,01; ***p ≤ 0,001; ****p ≤ 0,0001.

## Results

### Vaccine antigen design and optimization of the transgene expression cassette

The key target of rabies virus neutralizing antibodies is the G protein, which is the only surface-exposed protein on the virion particle. The nucleoprotein (N) is more conserved across lyssavirus species than the G protein [18] and stimulates both antibodies and T helper (Th) cell production [19]. Therefore, the G and N proteins may be suitable candidates for inclusion in rabies vaccines.

We have designed three synthetic vaccine antigens: one encoding only the rabies G protein (RG) and two composed of the G and N proteins in both combinations, RGN and RNG, as a single open reading frame with a self-cleaving Foot and Mouth Disease virus 2A sequence [20] between the two viral genes. The synthetic genes were codon-optimized for expression in human cells. The RG vaccine antigen was inserted in both the recombinant ChAd155 and ChAd83 vectors while the RGN and RNG vaccine antigens were inserted and evaluated only in the ChAd155 vector.

**Fig 1:**
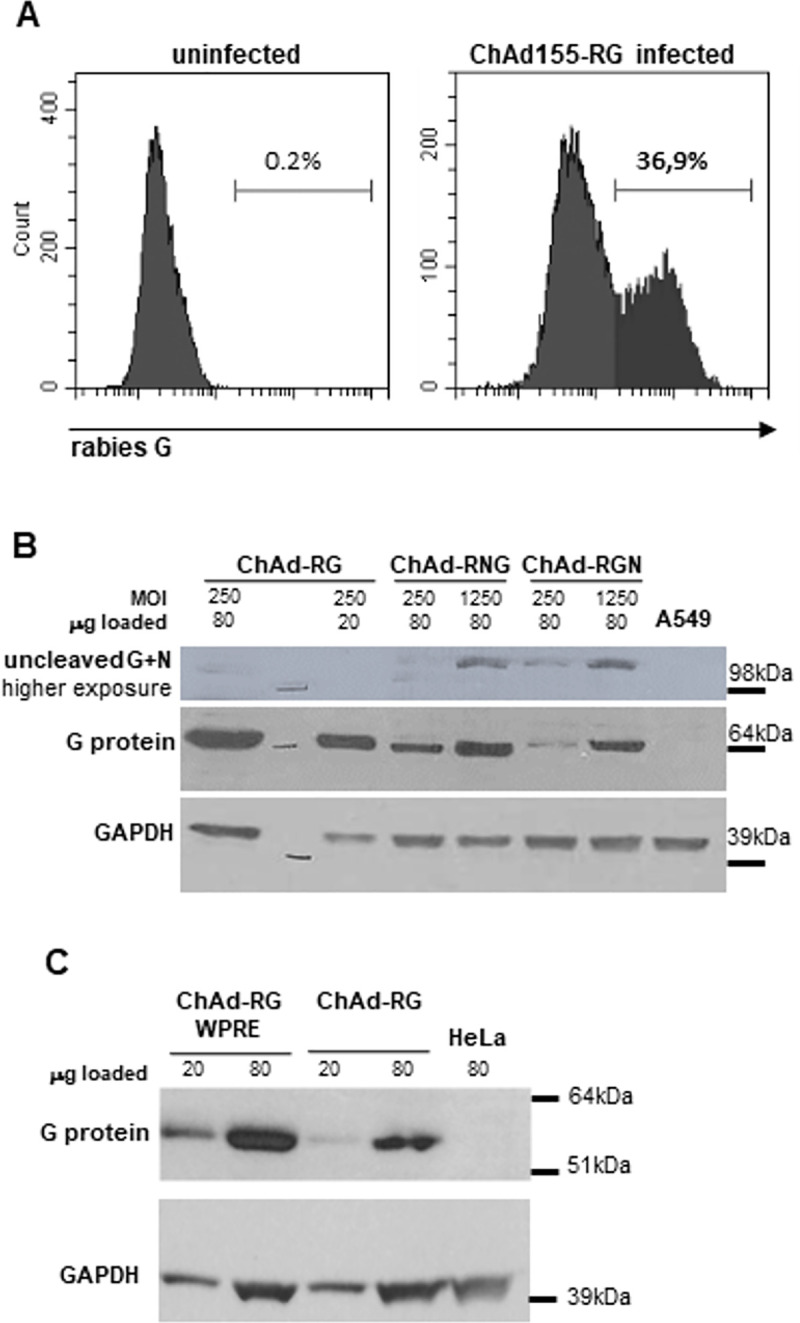
Rabies antigens expression in ChAd155 vectors. A) Whole cell FACS analysis of MRC5 cells not infected (left panel) and infected with 50 MOI (vp/cell) of ChAd155-RG (right panel). 48h after infection cells were stained with anti-Rabies Glycoprotein antibody and FITC-conjugated anti-mouse secondary antibody. B) Western blot analysis of G protein on lysates of A549 cells infected with 250 MOI of ChAd155-RG and 250 and 1250 MOI of ChAd155-RNG and ChAd155-RGN. 48h after infection cells were harvested and 20 μg and 80 μg of total cell lysates were used for WB analysis. GAPDH was used as a loading control. C) Effect of WPRE on G protein expression. Western blot analysis of G protein on total cell lysates. HeLa cells were infected with 250 MOI of ChAd155-RG +/- WPRE, 48h after infection cells were harvested and 20 μg and 80 μg of total cell lysates were analyzed by WB. GAPDH was used as a loading control.

The expression of the RG protein was assessed by whole-cell FACS analysis of human MRC5 cells infected with ChAd155-RG. Data shown in Fig 1A confirm that the protein is expressed and displayed on the cell membrane. To evaluate the expression of the RGN antigen, lysates of human A549 cells infected at different multiplicity of infection (MOI) with ChAd155-RG, ChAd155-RGN and ChAd155-RNG were run on a reducing SDS–PAGE and analyzed by Western blot (WB). As shown in Fig 1B, the lysates of ChAd155-RGN and ChAd155-RNG infected cells revealed a fragment consistent with the size of G protein, indicating that the precursor protein was correctly cleaved at the 2A site. Nevertheless, after longer exposure the lysates of ChAd155-RGN and ChAd155-RNG infected cells revealed also a band of higher molecular weight consistent with the size of the precursor protein, suggestive of an incomplete processing. Overall, the G protein expression levels were slightly inferior when G was fused to N.

Finally, we compared the level of G expression when the woodchuck hepatitis virus posttranscriptional regulatory element (WPRE), cloned upstream the polyadenylation site [21], was present or absent in the transgene expression cassette. As shown in Fig 1C, the addition of the WPRE clearly increased the steady-state expression levels of the RG protein.

### Immunogenicity and protective efficacy in mice identify ChAd155-RG as a suitable single-dose vaccine candidate

**Fig 2:**
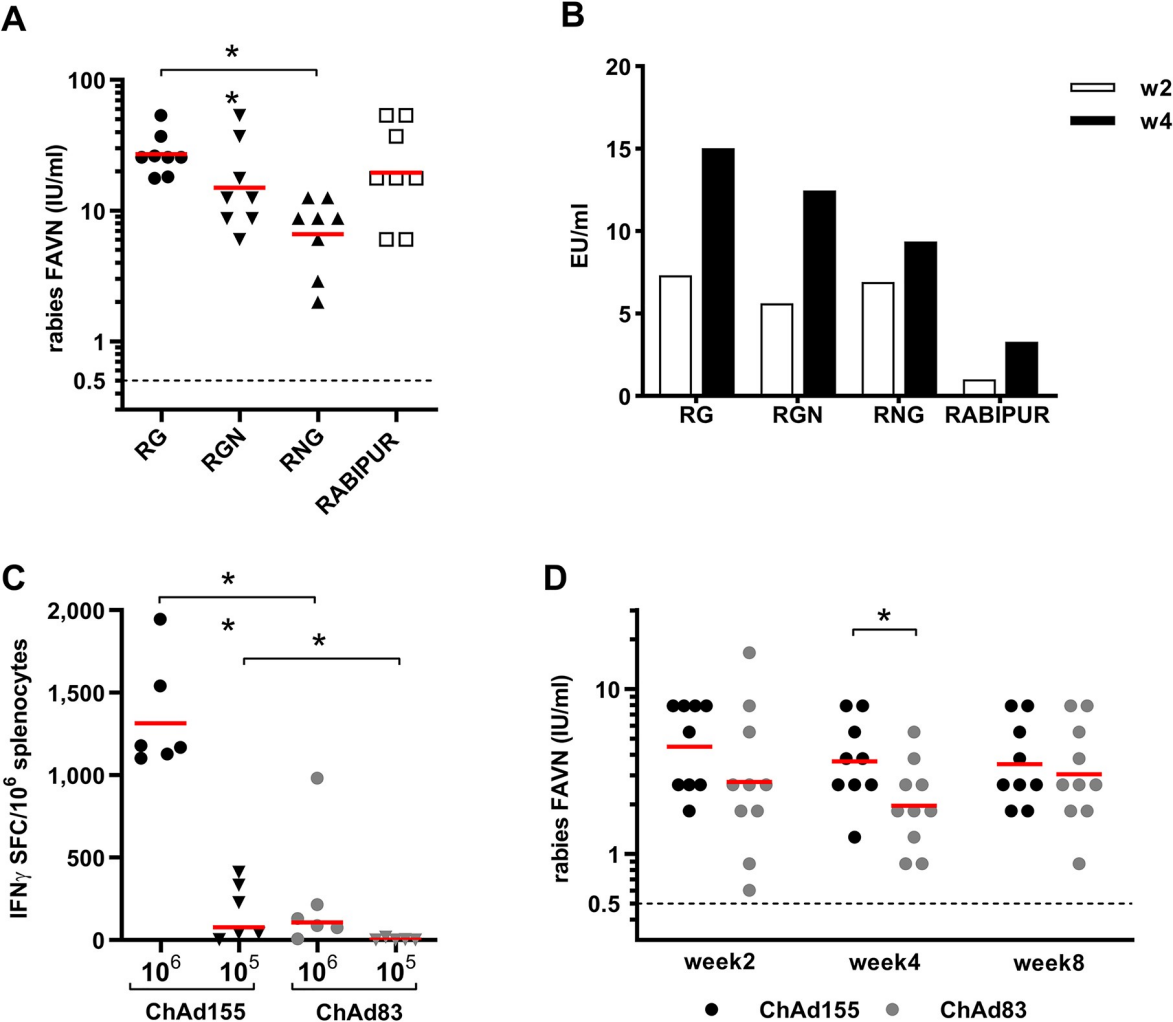
Immunological potency of ChAd155 and ChAd83 vectors in mice. A) Groups of CD1 mice (n = 8) were immunized with 1x10^8^ vp of ChAd155-RG, ChAd155-RGN and ChAd155-RNG, encoding different rabies antigen designs. As a control, one group of animals received 1/10th of the human dose of RABIPUR. Sera were collected 2 and 4 weeks after vaccination. Rabies VNA were measured by FAVN assay in sera collected at w4, and expressed as International Units (IU)/ml. B) Anti-G protein IgG measured by a commercially available ELISA kit (Platelia) at week 2 and 4 after immunization C) Groups of BALB/c mice (n = 8) were immunized with either 1x10^5^ or 1x10^6^ vp of ChAd155-RG, or ChAd83-RG. At 4 weeks after vaccination animals were sacrificed and T cell response measured in spleen of individual mice by IFNγ ELISpot assay. Data are expressed as number of IFNγ spot forming cells (SFC)/10^6^ splenocytes. D) BALB/c mice received 1x10^7^ vp of either ChAd155-RG, or ChAd83-RG, and were bled 2, 4 and 8 weeks after. Rabies VNA were measured by FAVN assay, and expressed as IU/ml. In all panels, the red lines represent group geometric mean.

To test the immunological potency of the different antigen combinations, the vaccine vectors ChAd155-RG, ChAd155-RNG and ChAd155-RGN were compared in a study conducted in outbred CD1 mice after intramuscular immunization at the dose of 1x10^8^vp/mouse. Outbred mice were selected for this type of study to better represent haplotype variability in human population and, based on previous experience, they mount more vigorous antibody responses compared to inbred mice. ChAd155 vaccines were administered at a dose of 10^8^ vp, which by dose/body weight ratio roughly corresponds to the target dose of 10^11^ vp in humans. RABIPUR was administered at 1/10^th^ of the human dose as a positive control for immunization. As shown in Fig 2A, rabies virus neutralizing antibody (VNA) titers measured by a standard Fluorescent Antibody Virus Neutralization (FAVN) test showed a trend for lower immunogenicity after vaccination with ChAd155-RGN and a statistically significant contraction of VNA titers with ChAd155-RNG. These data were confirmed using a commercially available ELISA kit (Platelia) to evaluate the titers of IgG binding to the rabies G protein in the sera of vaccinated animals (Fig 2B). Given the importance of functional antibodies directed to the G protein as correlate of protection against rabies, the vector expressing only the RG antigen was selected for further studies.

To develop a vectored vaccine suitable for a single-dose vaccination we have explored the immunological potency of the two novel chimpanzee-derived adenoviral vectors, ChAd155-RG and ChAd83-RG, in a dose–response intramuscular (IM) immunization study conducted in BALB/c mice. To better reveal any difference in potency for otherwise highly immunogenic adenoviral vectors, inbred mice were selected to avoid individual variability due to HLA haplotypes, and immunizations were conducted at low vector doses (10^6^ and 10^5^ vp for T cell responses, and 10^7^ vp for humoral response). We measured both VNA titers by FAVN assay at 8 and 12 weeks after vaccination and T-cell responses by IFN-γ ELISpot using overlapping 15-mer peptides spanning the RG antigen at 4 weeks after vaccination. The results shown in Fig 2C and 2D indicate that ChAd155-RG stimulates a significantly more potent cellular immunity than ChAd83-RG, and also indicate a trend for lower induction of VNA levels by the ChAd83 (group E) vaccine vector. Based on these results, ChAd155-RG was selected as the candidate single-dose rabies vaccine.

**Fig 3:**
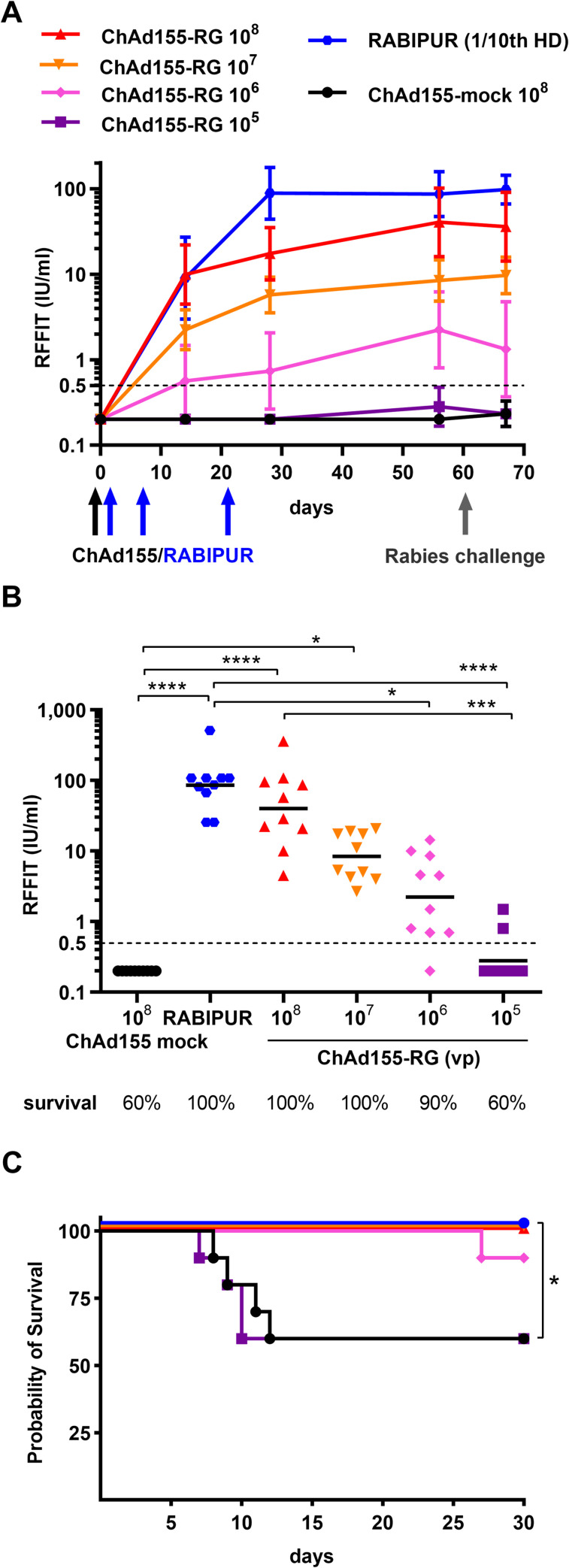
Protective efficacy and VNA magnitude and kinetics upon single vaccination with escalating doses of ChAd155-RG compared to three injections of RABIPUR. A) Mice (n = 10/group) were bled on day 0, 14, 28, 56 and 67. Serum from each animal was tested for detection of rabies VNA by RFFIT, and expressed as IU/ml. Data are shown as group geometric mean titer with 95% CI. Black and blue arrows indicate ChAd155 or RABIPUR (1/10th of the human dose, HD) vaccinations, while grey arrow indicates rabies challenge. B) VNA titers at day 56, three days before challenge, are shown for individual animals. Black lines correspond to geometric mean. For each group, the percentage of animals surviving 30 days post challenge is indicated at the bottom of the graph. C) Kaplan-Meier survival curves; log-rank Mantel-Cox test of the overall study: Chi square = 16.65, P = 0.0052; for mock vs RABIPUR, 10^8^ or 10^7^ vp comparisons: Chi square = 4.76, P = 0.029.

We next evaluated the protective efficacy of ChAd155-RG vaccine in outbred mice upon intramuscular challenge with a rabies virus strain. As shown in Fig 3A, a single injection of ChAd155-RG was able to elicit VNA titers above the threshold of 0.5 IU/ml over a wide dose range and with a clear dose effects; in addition, levels and duration of VNA induced by the single-dose vaccination with the vectored vaccine were not significantly inferior to those induced by 3 doses of RABIPUR (Fig 3A and 3B). Unexpectedly, the mortality observed with the control group receiving mock ChAd155 vaccination was lower than anticipated, based upon the prior challenge virus titration in naïve mice, and 4 out of 10 mice survived. Nevertheless, vaccination with ChAd155-RG conferred significant protection against RABV challenge at the two highest tested doses (Fig 3C), and no mice that developed titers ≥ 0.5 IU VNA succumbed to rabies virus challenge, irrespective of the immunization group (Fig 3B). All mice showing illness with signs of paralysis compatible with rabies virus infection following challenge were euthanized and demonstrated rabies virus antigens in their brain by the DFA test.

### Single-dose ChAd155-RG induces sustained rabies neutralizing antibodies in rabbits and non-human primates

Data gathered in mice showed that the vectored vaccine was protective and strongly immunogenic thus supporting ChAd155-RG as a suitable candidate for a single-dose vaccination against rabies. However, it is generally recognized that genetic vaccines are often less effective in large animals than in rodents (Gerdts et al., 2007). Therefore, the immunogenicity of the single-dose ChAd155-RG vaccine was explored in larger species, namely rabbits and non-human primates, which are a more relevant model for the development of a human vaccine and allow for delivery of human-dose levels.

In both species, we used 5x10^10^ vp of ChAd155-RG, corresponding to half of the intended human dose, and half human dose of the licensed RABIPUR vaccine.

**Fig 4:**
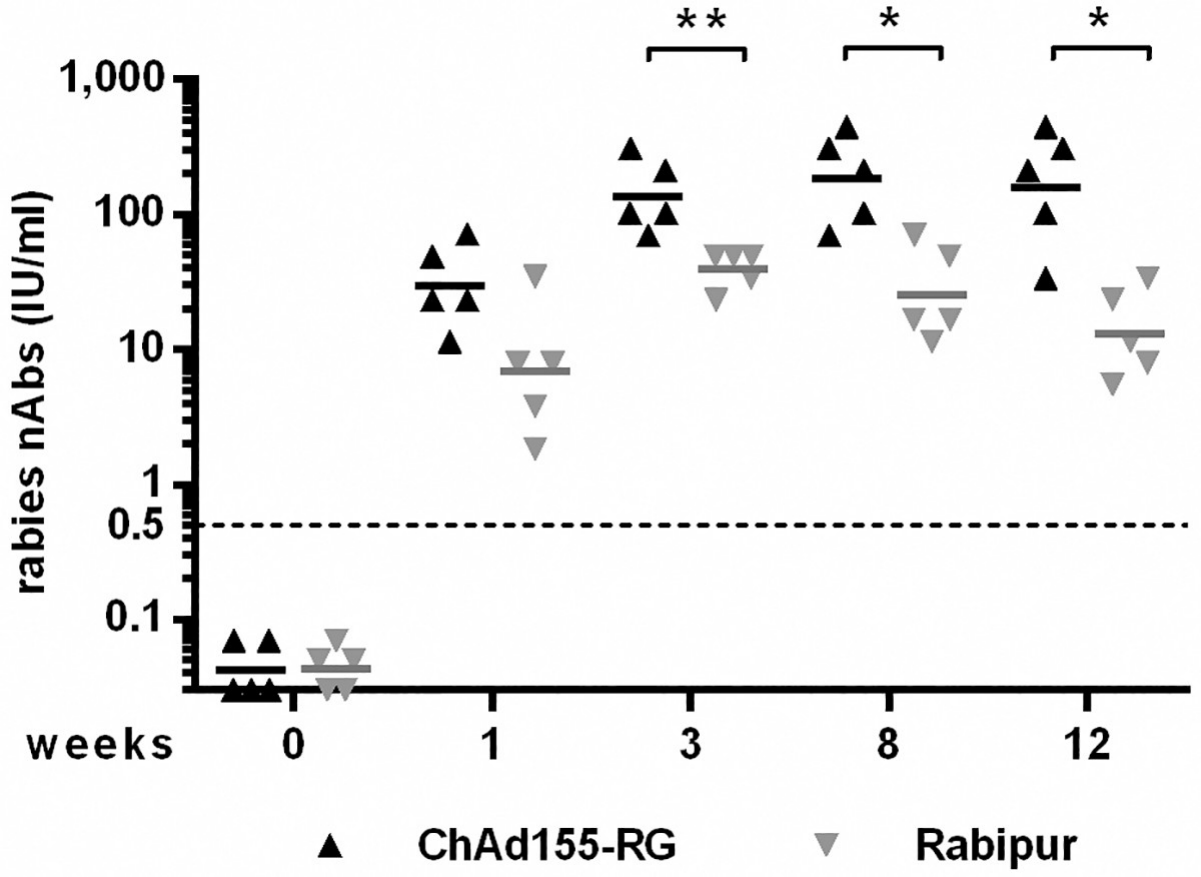
Kinetics of VNA induction after a single dose in rabbits. Groups of five rabbits each received a single intramuscular immunization with 5x10^10^vp of ChAd155-RG or 500μl RABIPUR (half human dose). Rabbits were bled on time of vaccination (week 0) and on weeks 1, 3, 8 and 12 post vaccination. Serum from each animal was tested for detection of rabies VNA by FAVN, and titers expressed as IU/ml. The horizontal lines represent geometric mean titers. A two-way analysis of variance was applied having as sources of variation “treatment” (the two vaccines), “time” (subsequent weeks of observation) and as dependent variable “antibody titer”. Statistically significant effect was found for both sources of variation and their interaction (treatment p = 0.0025, F (1, 8) = 18,78; time p = 0.011, F (2,043, 16,34) = 5,969; time x treatment p = 0.0118, F (4, 32) = 3,832).

A first study in rabbits was designed to compare the immunogenicity of ChAd155-RG and RABIPUR in a single dose regimen. ChAd155-RG was well tolerated in rabbits, with neither clinical signs nor local reactions at the injection site recorded in the 12 weeks of follow up. As shown in Fig 4, stronger and more durable VNA titers were elicited by the single dose IM vaccination with ChAd155 compared to the single dose of RABIPUR vaccine. Interestingly, animals receiving either vaccine showed very fast kinetics, with VNA titers well above the 0.5 IU/ml seroconversion threshold already one week after vaccination. The VNA titer declined significantly faster in animals receiving a single dose of RABIPUR respect to those immunized with ChAd155-RG, as determined by a two-way analysis of variance.

We next evaluated single ChAd155-RG administration to a full course of three RABIPUR injections in macaques, which allowed also a careful analysis of T cell responses.

**Fig 5:**
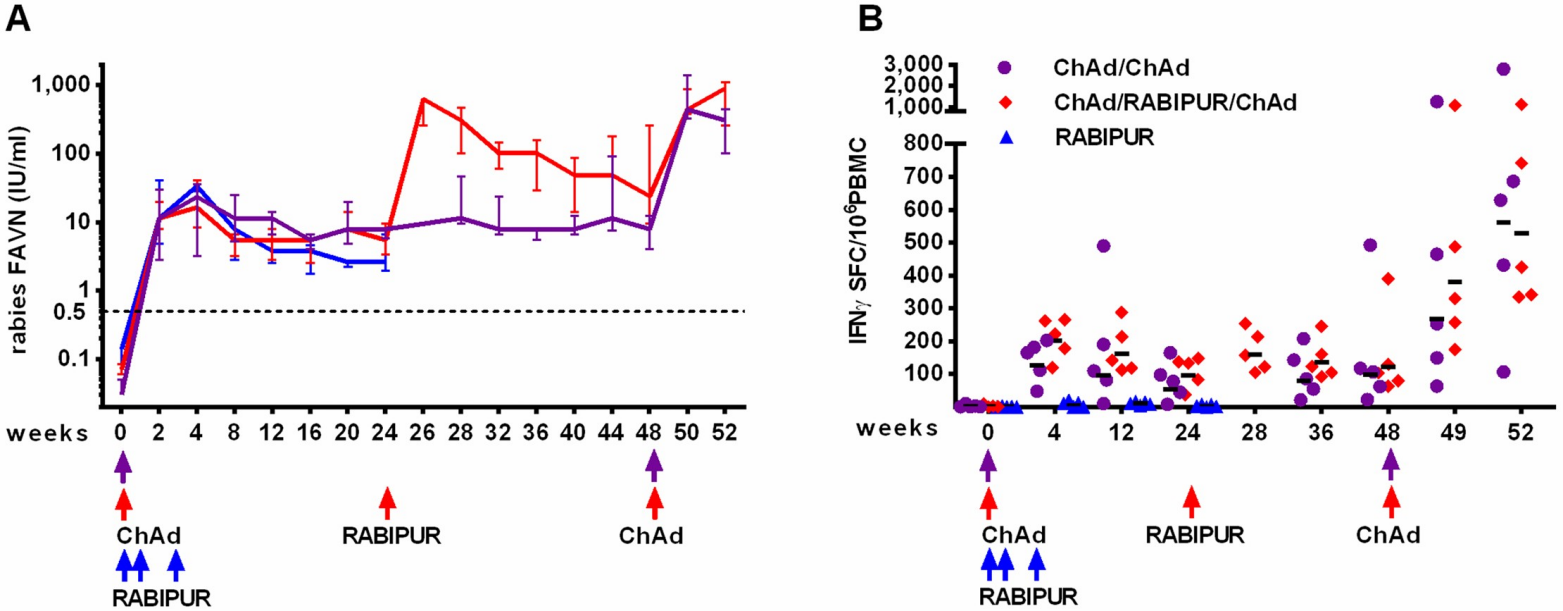
Rabies vaccine immunogenicity study in NHPs. Groups of 5 Cynomolgus monkeys were vaccinated intramuscularly as highlighted by arrows on x axis with 5x10^10^vp of ChAd155-RG vector (purple circles and red diamonds) and half human dose (500μl) RABIPUR (blue triangle). A: rabies VNA titer kinetics: Rabies VNA titers were measured in serum with a FAVN assay and followed starting at 2 weeks post vaccination and then monthly. Group median +/- IQR are shown. B: magnitude and kinetics of rabies-specific T cell response measured in PBMC at multiple time points by IFNγ ELISpot. Data are expressed as IFNγ SFC per million PBMC. Individual data points represent total rabies G response in each animal, obtained by summing reactivity to each of the 2 peptide pools covering rabies G protein and subtracting 2 times the DMSO background. Black lines represent geometric mean.

As shown in Fig 5A, vaccination with ChAd155-RG showed rapid kinetics of rabies VNA induction as measured by a standard FAVN assay, with titers above 0.5 IU/ml as early as 2 weeks after vaccination in all animals in groups 1 and 2; VNA titers peaked between week 2 and 4 and were of comparable magnitude to those induced by 3 doses of RABIPUR. Kinetics of contraction and steady-state levels were also similar between ChAd155-RG and RABIPUR vaccinated animals over the initial 6 months of follow up. Importantly, VNA titers remained stable and above 0.5 IU/ml up to week 48 (~1 year) after a single ChAd155-RG administration in all animals in group 1.

The vectored vaccine also induced rabies specific IFNγ secreting T cell responses, both CD8+ and CD4+ (Fig 5B and S1A Fig), which peaked around week 4 and then remained low but stable up to almost 1 year in group 1. Induction of T cell responses to the encoded transgene is a key feature of genetic vaccines and in fact, administration of 3 doses of RABIPUR did not elicit detectable IFNγ T cell responses to the G protein. IL4 was also monitored, as a marker of Th2 responses, but it was never detected at any time point (not shown).

### ChAd155-RG vaccination is compatible with the current indications for rabies post-exposure prophylaxis

To test the compatibility of ChAd155-RG with a licensed vaccine as a possible scenario of post-exposure prophylaxis in subjects previously immunized with the vectored vaccine, monkeys in group 2 received one dose of RABIPUR 6 months after ChAd155-RG administration. RABIPUR boost was indeed very efficient in raising VNA titers well above the peak level achieved after ChAd155-RG prime (Fig 5A), suggesting the full compatibility of the two rabies vaccine antigens (encoded G or inactivated virus). Finally, all animals in group 1 and 2 received a booster dose of 5x10^10^vp ChAd155-RG 48 weeks after first vaccination, mimicking a recall vaccination. ChAd155-RG boost at week 48 was also highly effective: VNA titers increased above the peak post-prime group 1 level, and at levels similar to those observed in group 2 upon RABIPUR boost. This highlights the possibility and potential efficacy of re-administering the candidate ChAd155-RG vaccine if a boost is needed (Fig 5A).

In contrast to VNA titres, T cell responses were poorly boosted by RABIPUR administration at 6 months in group 2 animals. However, ChAd155-RG re-administration at week 48 amplified rabies-specific T cell responses even beyond post prime levels (Fig 5B). Throughout the study we monitored induction and kinetics of neutralizing antibodies (nAb) against ChAd155 vector, which were readily induced upon first vector administration and declined over time (S1B Fig). Importantly, the long term boost with ChAd155-RG was highly effective in all 10 macaques even in presence of low to moderate ChAd155 nAb titers (mean titer at w48 of 239, range 31–586). Finally, IM administration of ChAd155-RG or RABIPUR or the combination of ChAd155-RG and RABIPUR was well tolerated in Cynomolgus monkeys. No clinical signs and no treatment-related effect on body weight were observed (not shown). Overall, these results confirmed the potential of ChAd155-RG as a single-dose vaccine for PrEP.

### ChAd155-RG remains confined to injection site and draining lymph nodes

**Fig 6:**
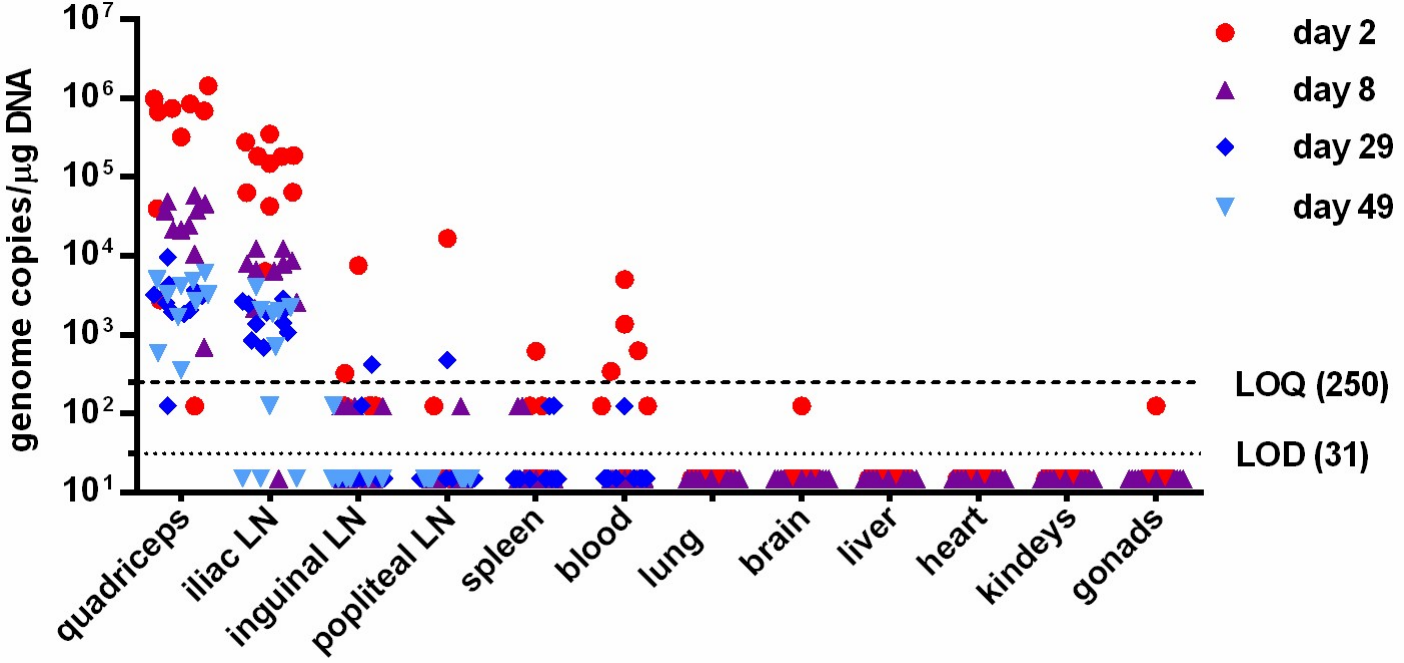
ChAd155-RG biodistribution in rats. ChAd155 genomes detected by qPCR in DNA extracted from indicated organs 2, 8, 29 and 49 days after intramuscular injection. Data are expressed as genome copies per mg of DNA. Limit of detection (LOD) and limit of quantification (LOQ), established within assay validation, were set at 12,5 and 100 copies/well respectively. For graphical representation, LOD and LOQ were adjusted to 1μg of tested DNA (31 and 250 copies respectively) and shown as dotted and dashed lines. Experimental samples resulting below LOD or LOQ were assigned values corresponding to one half of the extrapolated LOD and LOQ/mg of DNA (15 and 125 copies respectively).

A biodistribution study of ChAd155-RG after intramuscular administration in rats confirmed previous observations made with adenoviral vectors [22]. As shown in Fig 6, no viral DNA could be detected in brain, lung, heart, kidney, liver and gonads after vaccination, while in blood and spleen samples low levels of DNA were found in some animals only at day 2 post-administration, suggesting sporadic exposure possibly leaked from the injection site which was quickly cleared. Vaccine vector DNA was instead consistently detected at the injection site (quadriceps muscle) and in draining lymph nodes on the day of injection, which decreased over time but was still detectable at the end of the study period, 49 days after injection.

### Cross-neutralization of antibodies induced by vaccination with ChAd155-RG against different *Lyssavirus* species

Using the NHP vaccine study serum samples, we investigated the cross-neutralizing activity of the antibodies induced by ChAd155-RG and RABIPUR using pseudovirus-based neutralizing assays (PBNA) against the classical rabies vaccine strain CVS and other selected non-classical lyssavirus species of interest, namely EBLV-1 and EBLV-2, as well as MOKV, WCBV and IKOV.

**Fig 7:**
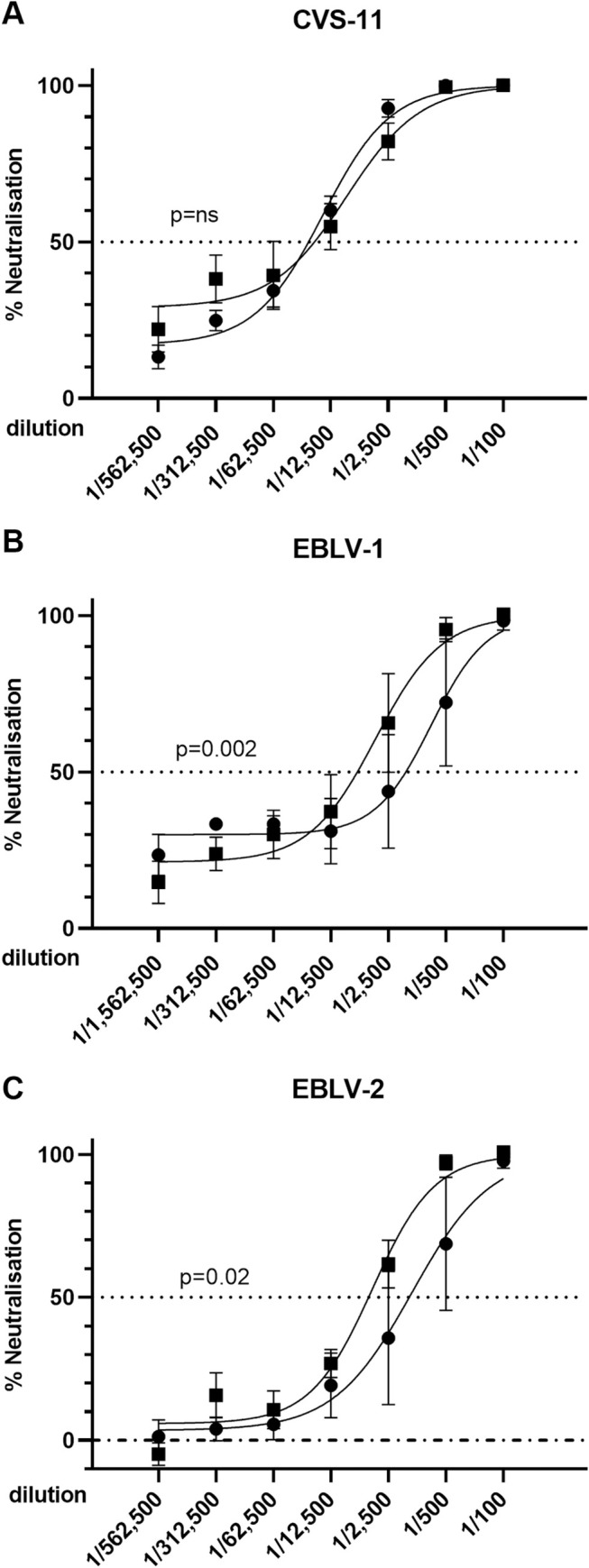
Neutralization of pseudoviruses harboring representative classical and non-classical phylogroup I lyssavirus glycoproteins. Immune sera obtained 5 weeks following a prime immunization of NHPs with either RABIPUR or ChAd155-RG were tested for their ability to neutralize pseudoviruses harboring either CVS-11 (A), EBLV-1 (B) or EBLV-2 (C) glycoproteins. Data represent the mean and standard deviation of combined normalized values for two replicate experiments, with each replicate experiment containing three technical replicates, and where 100% infectivity was set at the mean infectivity (measured in relative light units) observed in the absence of serum. Following non-linear regression analysis, an extra sum-of-squares analysis was performed to compare slopes and IC50 values. IC50 dilution were significantly higher for the ChAd155-RG serum than the RABIPUR serum, as indicated in each panel. RABIPUR (round symbols), ChAd155-RG (square symbols).

**Fig 8:**
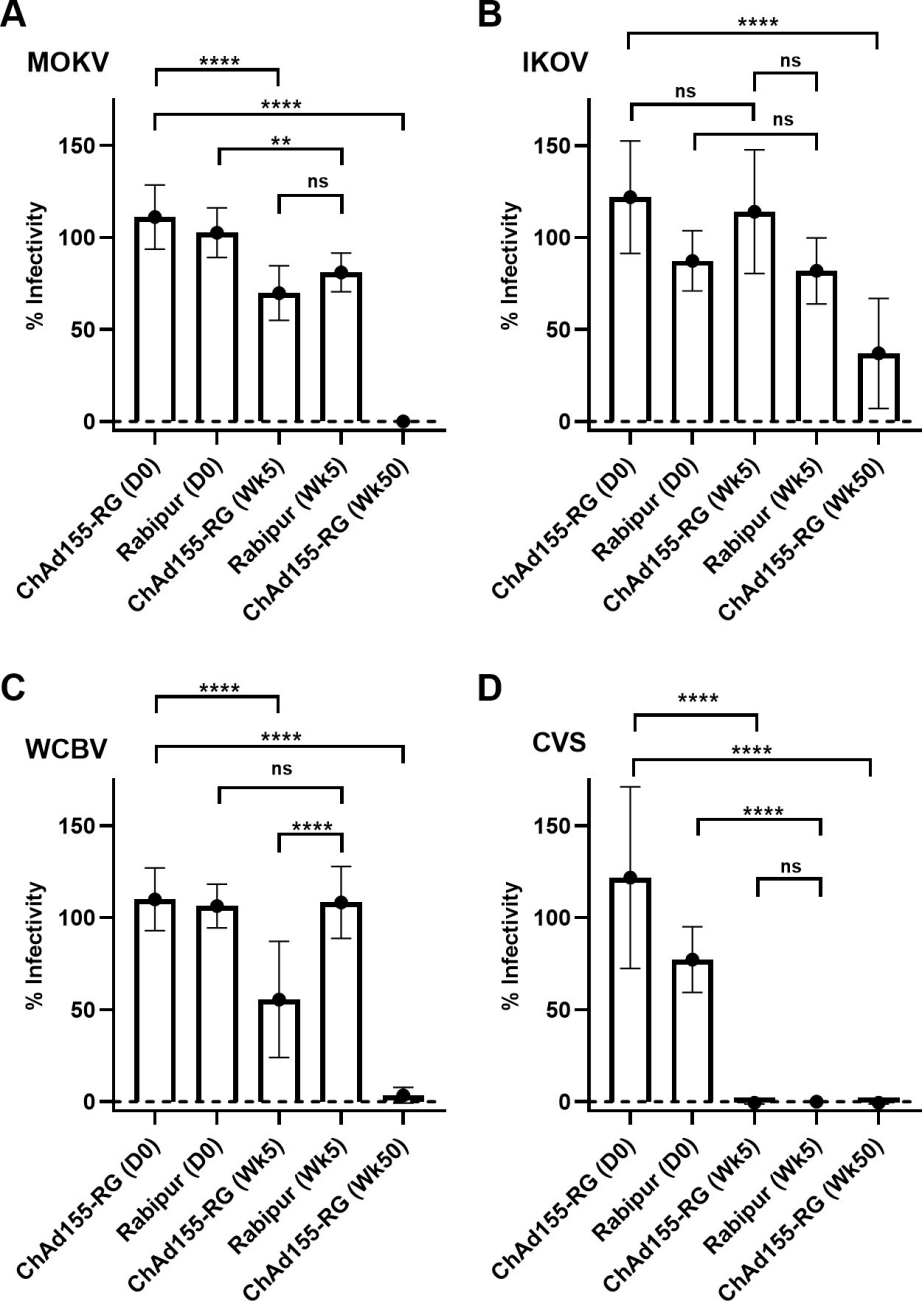
Neutralization of pseudoviruses harboring phylogroup II and III lyssavirus glycoproteins. Immune sera obtained 5 weeks following immunization of NHPs with either RABIPUR or ChAd155-RG, and at week 50 following a ChAd155-RG boost were tested for their ability to neutralize pseudoviruses harboring either MOKV (A), IKOV (B), WCBV (C) or control classical CSV-11 (D) glycoproteins. Data represent the mean and standard deviation of combined normalized values for two replicate experiments, with each replicate experiment containing three technical replicates, and where 100% infectivity was set at the mean infectivity (measured in relative light units) observed in the absence of serum.

Serum samples obtained after vaccination with a single dose of ChAd155-RG led to comparable dose-response curves against CVS-11 to those obtained following a full RABIPUR vaccination course (Fig 7A). The non-classical, phylogroup I lyssaviruses EBLV-1 and EBLV-2 were neutralized with less efficiency than CVS-11, and in general lower concentrations of ChAd155-RG serum were required for neutralization compared to serum elicited by RABIPUR, (Fig 7B and 7C) and this difference reached statistical significance in a comparison of mean IC_50_ values. Neutralization curves for individual animals are presented in S2A–S2C Fig. To assess whether the neutralization observed extended to phylogroup II and III lyssaviruses a single serum dilution neutralization comparison was performed (Fig 8). For the RABIPUR vaccinated animals, the only pseudovirus that showed significant levels of neutralization compared to the day zero control was the MOKV lyssavirus (Fig 8A), as well as the control group 1 CSV-11 virus (Fig 8D). By comparison the week 5 serum from animals vaccinated with ChAd155-RG showed significant neutralization against MOKV, WCBV and CSV-11, and the neutralization potency against all viruses showed a significant increase following the boost at week 48, including potent neutralization of the phylogroup III virus IKOV (Fig 8B). To demonstrate vaccine-specific and dose-dependent neutralization of the phylogroup II MOKV virus, a single dilution series was performed for week 5 serum obtained from the ChAd155-RG and by RABIPUR vaccinated animals using the MOKV pseudovirus (S2D Fig) and for sera obtained from two animals receiving a ChAd155-RG boost at 48 weeks (S2E Fig).

## Discussion

Unlike other viruses targeted for elimination, it is very unlikely that rabies will be eradicated because of the presence of lyssaviruses in bats [23]. The realistic aim in the 21st century is to enhance efforts towards the elimination of canine-mediated human rabies by 2030 [24]; a goal that has already been achieved in several regions [25] [26]. The Philippines is currently the only country that has introduced wide-scale pre-exposure vaccination of children in highly endemic areas of canine rabies. The ‘anti-rabies act’, signed into law with the objective of human rabies elimination by 2020, provides free routine PrEP to schoolchildren aged 5–14 years in areas where rabies incidence exceeds 2.5 human rabies cases per million population [27]. The effectiveness of more wide-spread PrEP has been validated in Peru [28]. This country has successfully stopped the increasing incidence of vampire bat-transmitted rabies in children in Amazonia by incorporating the rabies vaccine into routine childhood immunization programs. Nevertheless, the use of current rabies vaccines for childhood PrEP is not cost-effective [29], and necessitates the development of cheaper and more immunogenic single dose vaccines that are stable and that preferably would not require a cold-chain.

To achieve these objectives, many alternative rabies vaccines are in the experimental phase of development, such as DNA and RNA based vaccines, attenuated RABV, plant-based vaccines and recombinant virus-vector vaccines [30]. To date, only a mRNA- based vaccine has progressed to Phase I clinical trial [31] but, despite the repeated doses, the mRNA vaccine was clearly inferior to licensed vaccines and requires further development of better optimized formulations.

Adenovirus vectors are among the safest and most immunogenic of the different types of viral vector vaccines [32]. Replication-defective, non-human serotypes have been vectored [33], to overcome potential dampening of vaccine immunogenicity due to pre-existing neutralizing antibodies to human adenoviruses [8]. A group E simian adenovirus vector AdC68.010.rabgp expressing the rabies virus glycoprotein was shown to induce long-lasting seroconversion after a single dose in non-human primates [34]. More recently this vector has been further modified to improve productivity and transgene expression [35]; the improved candidate, ChAdOx2 RabG, has shown higher VNA induction than AdC68.010.rabgp when administered at low dose in mice.

Here we present a novel candidate single-dose adenovirus rabies vaccine, which consists of a group C simian adenovirus vector, ChAd155-RG expressing a codon optimized rabies virus medoid glycoprotein. We show that ChAd155-RG vector, belonging to group C, is a more potent vaccine than a group E vector, ChAd83-RG. Both vectors contain equivalent deletions of early viral genes and identical transgene expression cassettes. Despite showing similar G protein expression levels in infected cultured cells (S3 Fig), ChAd155-RG induces stronger rabies specific T cells and moderately higher VNA titers in mice vaccinated with equivalent doses of ChAd83-RG. However, it has been reported that different adenoviral vectors show different levels of antigen expression *in vivo* [36, 37], together with different innate immunity profiles [38] [39], suggesting that these parameters might affect each other and shape the resulting adaptive immune response. Alternatively, host-dependent differences in the immune responses to different vaccine vectors could explain the results obtained in mice; indeed, it has been reported that two group E vectors, ChAdOx1 and AdC68, were immunologically less potent than the group C human Ad5 in mice but showed equivalent, if not stronger immune responses in cattle [40]. Given the high productivity of ChAd83-RG, this vector would deserve further evaluation in a more relevant animal species or a direct comparison with ChAd155-RG in a Phase 1 clinical study.

A number of pieces of evidence in the literature suggests that both the G and N proteins may be suitable candidates for inclusion in rabies vaccines [41]. N protein stimulates T cell production [19] and, being more conserved than G across different species, induces cross-protection against intramuscular rabies challenge [42]. The mechanism of protection against rabies virus challenge in the absence of VNA could be attributed to the induction of cytolytic T cells as well as T helper cells that support the activity of VNA-producing B cells, or it could act by promoting the attachment of anti-N antibody via the Fc receptor to phagocytic cells, which are then stimulated by the infecting challenge virus to produce cytokines that inhibit viral replication [43]. Finally, it has been shown that N protein stimulates VNA production induced by classical vaccines [42]. Despite this evidence, our attempt to include the N protein in the vaccine transgene together with the G protein as a single open reading frame with a self-cleaving 2A sequence resulted in decreased anti-G IgG titer, as detected by a commercial ELISA (Platelia), and decreased VNA stimulation *in vivo* compared to the vaccine expressing the G protein alone. This could in part be explained by an inefficient processing of the precursor fusion protein, as revealed by Western blot analysis of G protein expression in ChAd155-RGN infected cells. However, since the immunization with the vector ChAd155-RG expressing only the G protein was effective at inducing rabies specific T cell responses, we considered inclusion of the N antigen in the vaccine to be dispensable. Notably, we observed that the relationship between ELISA-measured antibody and VNA titers differed for ChAd155-RG and RABIPUR vaccines. Such difference was already described by Wang et al [35] for a different simian adenoviral vector, ChAdOx2 RabG, when compared to an inactivated rabies vaccine. A reasonable explanation for the stronger induction of anti-G antibodies by the vectored vaccines is that epitopes displayed to B cells by vector-infected cells may be different from those displayed by rabies virions in both spatial arrangement and, perhaps, steric accessibility of membrane-proximal regions. Here we present evidence that a single dose of ChAd155-RG is highly immunogenic in mice and shows protective efficacy at the highest doses upon challenge with a bat-derived rabies virus strain. However, a strong limitation of this study was the unexpected low mortality observed in the control group.

It has recently been shown that in mice immunized with RABV-G mRNA, CD4 T cells are crucial for the generation of neutralizing antibodies [44]. In fact, CD4-depleted mRNA-vaccinated mice showed VNA titers more than 15-fold reduced, compared to the CD4-intact mRNA-vaccinated group. Consistently, all mice in the CD4-depleted, mRNA vaccinated group had to be sacrificed within 11 days after rabies challenge, while all animals survived in the CD4-intact mRNA-vaccinated group [44]. Indeed, the help of rabies specific T cells induced by ChAd155-RG could explain the more sustained VNA levels achieved by the single-dose vaccine vector respect to the single-dose RABIPUR vaccination in rabbits.

Notably, we have observed very rapid kinetics of induction of VNA by ChAd155-RG in both mice, rabbits and NHPs. In vaccinated NHPs VNA levels peaked well above the seroconversion threshold in all animals between two and four weeks after the single-dose administration; at two months VNA titers slightly declined but then remained sustained for the whole year of follow up. The very fast onset of VNA induced by the vectored vaccine, which is almost superimposable with the VNA kinetics induced by the 3 repeated doses of RABIPUR, identifies ChAd155-RG as a suitable vaccine also for PEP, where the VNA serum threshold of 0.5 IU/ml must be achieved in two weeks after vaccination [7].

The fast kinetics of VNA induction observed with ChAd155-RG are in contrast with those observed in NHPs vaccinated with AdC68.010.rabgp [34], where VNA titers peaked 2 months after vaccination and, as a consequence, the vaccine was not protective in PEP after rabies challenge. The same kinetics of VNA induction were measured after immunization with 10^9^ or 10^11^vp of AdC68.010.rabgp, ruling out the possibility that a different dose could be the reason for the faster kinetics observed with ChAd155-RG vaccine. A possible explanation for the different kinetics could still be the genetic distance between ChAd155-RG and AdC68.010.rabgp adenovirus vectors, which belong to group C and group E (similar to ChAd83), respectively, or due to the different transgene expression cassettes. Conversely, both ChAd155-RG and AdC68.010.rabgp vectored vaccines showed sustained VNA titers over several months of follow up. Such a feature could be the consequence of the persistence of adenovirus genomes in a transcriptionally active form. Replication-defective adenovirus vectors are not cytolytic and have been shown to persist at low levels in a transcriptionally active form for an extended period of time [10], thus constantly providing a boost to the immune system. Immune responses to adenovirus vector-encoded transgenes thus remain stable over long periods of time [10]. Consistently, we have demonstrated that viral DNA is still detectable in the muscle and in draining lymph nodes 49 days after intramuscular immunization with ChAd155-RG in a biodistribution study in rats. Importantly, we demonstrate here that the vectored vaccine is compatible with the licensed vaccine in a simulated PEP in NHP, since it strongly boosts VNA titers induced by a previous vaccination with RABIPUR. In addition, a homologous boost with ChAd155-RG one year after priming was highly effective in all animals, despite the presence of anti-vector nAb titers similar to those detected in humans with pre-existing cross-reactive immunity to ChAd155 [45]. On the basis of genetic distance and serological cross-reactivity, the Lyssavirus genus has been subdivided into three broad phylogroups I-III. Of the known lyssaviruses, only six species (RABV, EBLV-1, EBLV-2, ABLV, DUVV, IRKV) in phylogroup I and MOKV in phylogroup II have caused documented human deaths [6]. The remaining species of the genus, WCBV, cannot be included in either phylogroups I and II and is suggested to be considered a representative of an independent phylogroup III. Another divergent lyssavirus, related phylogenetically to WCBV (therefore potentially a member of the proposed phylogroup III) named IKOV, was detected in an African civet in the United Republic of Tanzania. Modern rabies vaccines produced and used worldwide are ineffective against infection with lyssaviruses belonging to phylogroups II-III [6]. Recently, the purified chick embryo vaccine (PCEC) Flury strain of RABV (RABIPUR^)^ has been shown to induce antibodies that fully neutralize EBLV-1, EBLV-2, ABLV, BBLV and DUVV (all phylogroup I) and partially cross-neutralize MOKV, a more distant species belonging to phylogroup II [46]. The RABV medoid G protein cloned in the ChAd155 vectored vaccine is approximately 94% identical to that of the RABV strains used in modern vaccines, while having a lower percentage of identity to the other species (DUVV, EBL-1, EBL-2) in phylogroup I that causes death in humans. Using a pseudovirus-based neutralization assay, we show that antibodies induced in macaques by a single dose of ChAd155-RG show neutralizing activity against RABV, EBLV-1 and EBLV-2 which appears similar or, in the case of EBLV-1 and EBLV-2, superior to that induced by a full course of RABIPUR vaccine. The single primer dose of ChAd155-RG also demonstrated superior neutralization to the divergent lyssavirus WCBV. Potency and breadth was extended to the highly divergent lyssavirus IKOV following the week 48 boost. Indeed, the described partial neutralization of MOKV strain by RABIPUR vaccine was observed on sera of individuals boosted with RABIPUR at various times after the completion of a full course of PrEP, in a so called ‘simulated PEP’ study [46], thus suggesting that high VNA titers induced by the vectored vaccine after boosting could be functionally similar to those induced by RABIPUR after a full course of simulated PEP. However, an obvious limitation of our studies is the low numbers of analyzed sera, which doesn’t allow to draw conclusions on the superiority of ChAd155-RG versus RABIPUR vaccination. These observations need to be confirmed and expanded in future studies using larger number of sera from vaccinated animals.

In conclusion, we have presented evidence that a single-dose chimp adenovirus vectored rabies vaccine, ChAd155-RG, can rapidly induce serum VNA titers and T cell responses, which are sustained over time and can be effectively boosted by a licensed vaccine. This next-generation vaccine could address the different attributes required to protect humans from rabies disease in both PrEp and PEP, thus improving compliance and enhancing successful implementation of rabies control programs.

## Supporting information

S1 Fig Complementary immunogenicity data in NHPs. A: IFNγ intracellular staining (ICS) and FACS analysis of PBMC from animals with IFNγ ELISpot responses above 200 SFC/million PBMC at three time points. Data are expressed as the percentage of CD8 or CD4 secreting IFNγ upon stimulation with rabies G peptide pools. Lines represent geometric mean. B: ChAd155 neutralizing antibody titers measured in serum at multiple time points by means of a Secreted Alkaline Phosphatase (SEAP) assay. The neutralization titer is defined as the reciprocal of sera dilution required to inhibit SEAP expression by 50% compared to the SEAP expression of virus infection alone. Titers for individual animals (symbols) and mean with SD are shown for each time point. Arrows indicate when animals received ChAd155-RG vaccinations. The dashed line at 18 indicate assay cutoff, corresponding to the lowest serum dilution point tested.
(TIF)

S2 Fig Neutralization of pseudoviruses harboring representative classical, non-classical and Phylogroup II lyssavirus glycoproteins. Immune sera obtained 5 weeks following immunization of individual NHPs with either RABIPUR or ChAd155-RG were tested for their ability to neutralize pseudoviruses harboring either CVS-11 (A), EBLV-1 (B) or EBLV-2 (C) glycoproteins. To investigate antibody breadth across a dilution range, pseudoviruses containing glycoprotein from the Phylogroup II MOKV were neutralized with sera obtained 5 weeks following immunization of individual NHPs with either RABIPUR or ChAd155-RG (D) and with sera obtained from two animals receiving a ChAd155-RG boost at 48 weeks (E). RABIPUR (green symbols), ChAd155-RG (blue symbols and each symbol/line represents serum dilutions/tests from a single animal.
(TIF)

S3 Fig Western Blot analysis of G protein on total cell lysates. HeLa cells were infected with 50, 250 and 1250 MOI (vp/cell) of both ChAd155-RG and ChAd83-RG and 48h after infection cells were harvested for total protein extraction. 50 μg of total cell lysates was used for WB analysis.
(TIF)
