# Correction: A next generation vaccine against human rabies based on a single dose of a chimpanzee adenovirus vector serotype C

**DOI:** 10.1371/journal.pntd.0009348

**Published:** 2021-04-13

**Authors:** Federico Napolitano, Rossella Merone, Adele Abbate, Virginia Ammendola, Emma Horncastle, Francesca Lanzaro, Marialuisa Esposito, Alessandra Maria Contino, Roberta Sbrocchi, Andrea Sommella, Joshua D. Duncan, Joseph Hinds, Richard A. Urbanowicz, Armin Lahm, Stefano Colloca, Antonella Folgori, Jonathan K. Ball, Alfredo Nicosia, Benjamin Wizel, Stefania Capone, Alessandra Vitelli

The twelfth author’s name is spelled incorrectly. The correct name is: Joseph Hinds.
