# Supplementary figures and images for: A next generation vaccine against human rabies based on a single dose of a chimpanzee adenovirus vector serotype C
Source: PLoS Negl Trop Dis. 2020 Jul 15;14(7):e0008459. doi: 10.1371/journal.pntd.0008459 (PMC7363076; doi:10.1371/journal.pntd.0008459)

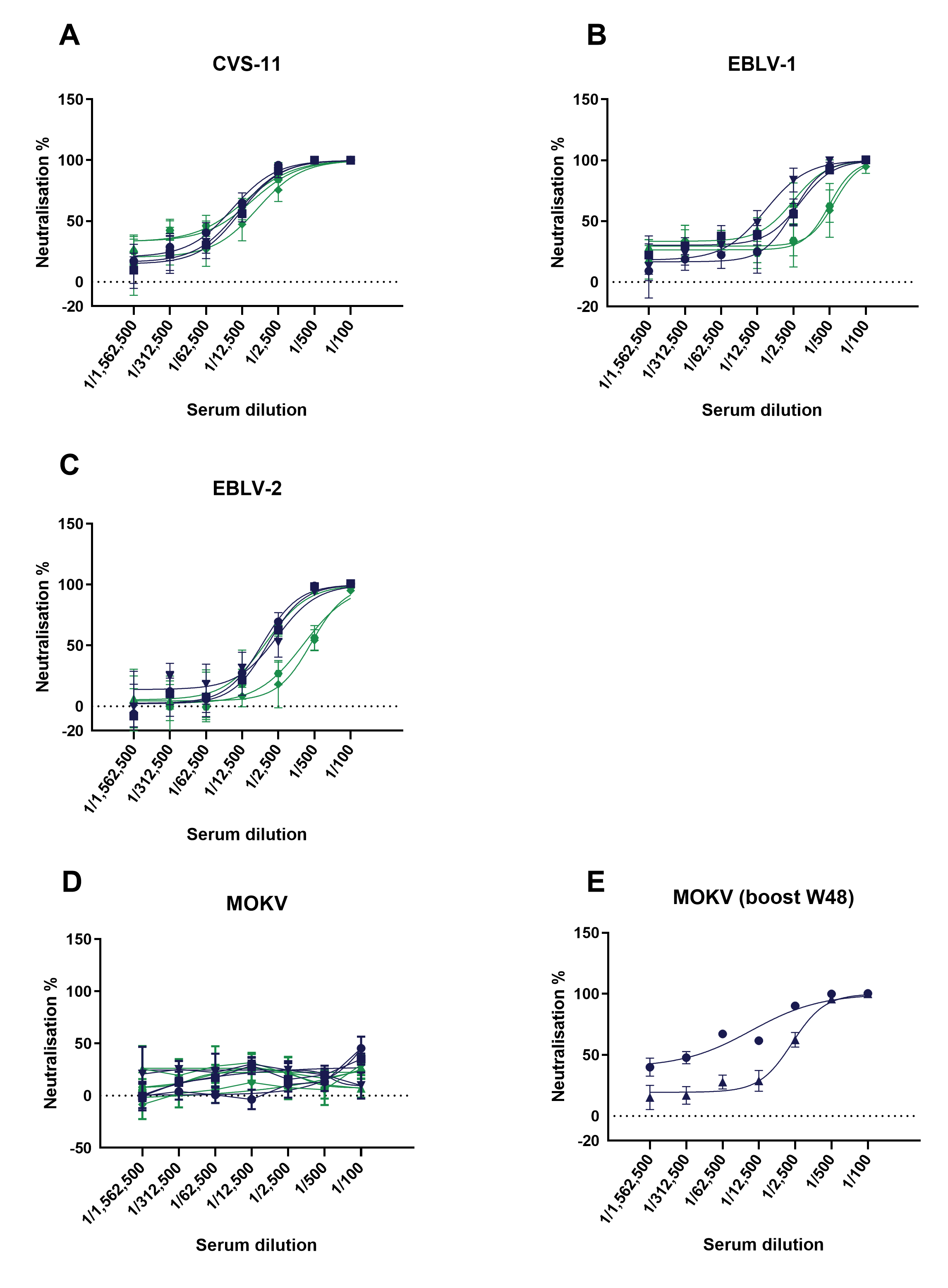

Supplement: S2 Fig — Immune sera obtained 5 weeks following immunization of individual NHPs with either RABIPUR or ChAd155-RG were tested for their ability to neutralize pseudoviruses harboring either CVS-11 (A), EBLV-1 (B) or EBLV-2 (C) glycoproteins. To investigate antibody breadth across a dilution range, pseudoviruses containing glycoprotein from the Phylogroup II MOKV were neutralized with sera obtained 5 weeks following immunization of individual NHPs with either RABIPUR or ChAd155-RG (D) and with sera obtained from two animals receiving a ChAd155-RG boost at 48 weeks (E). RABIPUR (green symbols), ChAd155-RG (blue symbols and each symbol/line represents serum dilutions/tests from a single animal. (TIF) [file pntd.0008459.s002.tif]

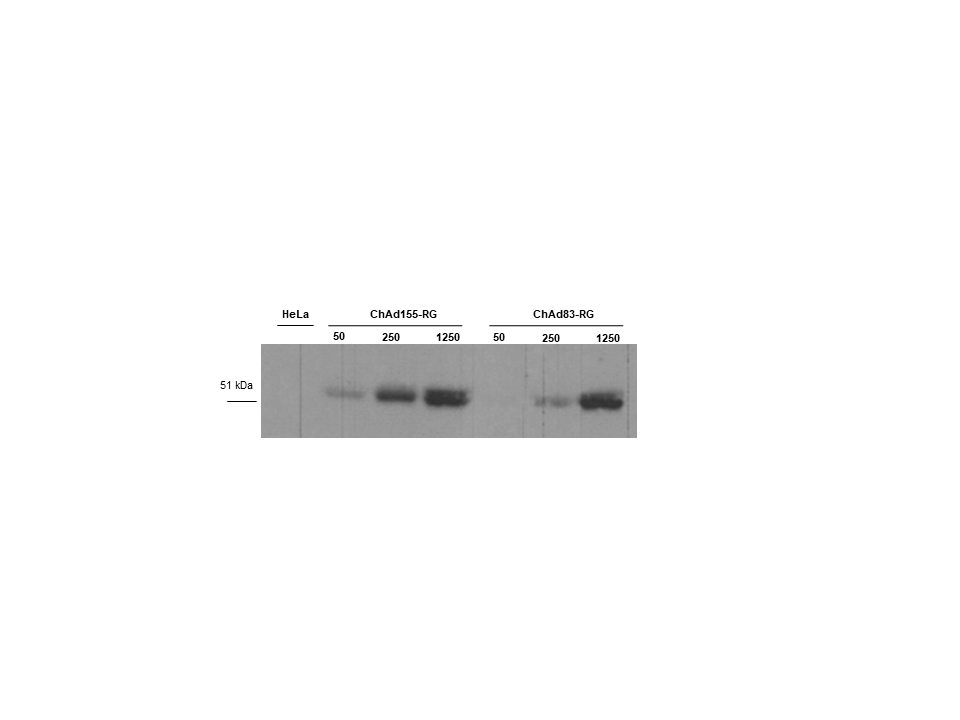

Supplement: S3 Fig — HeLa cells were infected with 50, 250 and 1250 MOI (vp/cell) of both ChAd155-RG and ChAd83-RG and 48h after infection cells were harvested for total protein extraction. 50 μg of total cell lysates was used for WB analysis. (TIF) [file pntd.0008459.s003.tif]
